# Rodent models to study the metabolic effects of shiftwork in humans

**DOI:** 10.3389/fphar.2015.00050

**Published:** 2015-03-24

**Authors:** Anne-Loes Opperhuizen, Linda W. M. van Kerkhof, Karin I. Proper, Wendy Rodenburg, Andries Kalsbeek

**Affiliations:** ^1^Department of Hypothalamic Integration Mechanisms, Netherlands Institute for Neuroscience, Hypothalamic Integration MechanismsAmsterdam, Netherlands; ^2^Centre for Health Protection, National Institute for Public Health and the EnvironmentBilthoven, Netherlands; ^3^Centre for Nutrition, Prevention and Health Services, National Institute for Public Health and the EnvironmentBilthoven, Netherlands; ^4^Department of Endocrinology and Metabolism, Academic Medical Center, University of AmsterdamAmsterdam, Netherlands

**Keywords:** shiftwork, metabolism, animal model, circadian desynchronization, glucose, lipids, activity, obesity

## Abstract

Our current 24-h society requires an increasing number of employees to work nightshifts with millions of people worldwide working during the evening or night. Clear associations have been found between shiftwork and the risk to develop metabolic health problems, such as obesity. An increasing number of studies suggest that the underlying mechanism includes disruption of the rhythmically organized body physiology. Normally, daily 24-h rhythms in physiological processes are controlled by the central clock in the brain in close collaboration with peripheral clocks present throughout the body. Working schedules of shiftworkers greatly interfere with these normal daily rhythms by exposing the individual to contrasting inputs, i.e., at the one hand (dim)light exposure at night, nightly activity and eating and at the other hand daytime sleep and reduced light exposure. Several different animal models are being used to mimic shiftwork and study the mechanism responsible for the observed correlation between shiftwork and metabolic diseases. In this review we aim to provide an overview of the available animal studies with a focus on the four most relevant models that are being used to mimic human shiftwork: altered timing of (1) food intake, (2) activity, (3) sleep, or (4) light exposure. For all studies we scored whether and how relevant metabolic parameters, such as bodyweight, adiposity and plasma glucose were affected by the manipulation. In the discussion, we focus on differences between shiftwork models and animal species (i.e., rat and mouse). In addition, we comment on the complexity of shiftwork as an exposure and the subsequent difficulties when using animal models to investigate this condition. In view of the added value of animal models over human cohorts to study the effects and mechanisms of shiftwork, we conclude with recommendations to improve future research protocols to study the causality between shiftwork and metabolic health problems using animal models.

## Introduction

Our current 24-h society requires an increasing number of employees to work nightshifts and as a result millions of people worldwide work during the evening or night for a certain period during their life. In the Netherlands, 16% of the working population works regularly or occasionally during the night, whereas 51% of the population sometimes or regularly works during the evening (Centraal Bureau voor de Statistiek, 2013). Epidemiological studies show correlations between shiftwork and an increased risk of cancer, cardiovascular disease, sleep disturbances, impaired psychosocial health and gastrointestinal problems (Matheson et al., 2014). Moreover, the last two decades, population-based studies have shown that there is also an association between shiftwork and development of metabolic problems, including metabolic syndrome (Van Amelsvoort et al., 1999; Karlsson et al., 2001, 2003; Biggi et al., 2008; Suwazono et al., 2008; Lin et al., 2009; Pietroiusti et al., 2010; Kubo et al., 2011; Li et al., 2011; Tucker et al., 2012; Ye et al., 2013; Kawabe et al., 2014; Kawada and Otsuka, 2014), altered glucose metabolism (De Bacquer et al., 2009; Suwazono et al., 2009; Oyama et al., 2012), altered lipid metabolism (Biggi et al., 2008; De Bacquer et al., 2009; Dochi et al., 2009) and high blood pressure (Morikawa et al., 1999; Sakata et al., 2003; De Bacquer et al., 2009; Lin et al., 2009). Population-based studies are limited in their use for understanding causality and underlying mechanisms to explain the relationship between shiftwork and disease. Using experimental studies in humans is problematic due to the fact that many metabolic outcomes, such as body weight and composition, are long-term effects. Certainly, acute effects of shiftwork conditions on metabolic parameters can be studied in humans, which is currently done (McHill et al., 2014). Therefore, animal studies have been used to gain more insight in these questions. In the current review we provide an overview of the different animal models that are available to investigate the mechanism underlying the negative health consequences of shiftwork.

Daily 24-h rhythms are present throughout the body's physiology and can be observed in, for example, sleep, food consumption, body temperature and numerous hormone levels (Dibner et al., 2010). These rhythms are regulated by the central circadian clock in the suprachiasmatic nucleus (SCN) of the hypothalamus and circadian oscillators in peripheral tissues and organs (the so-called peripheral clocks). The endogenous rhythmicity of the SCN neurons ultimately results from the interaction between a set of rhythmically expressed genes, so-called clock genes, which are expressed in almost every cell of the body. In the SCN, the nearly 24-h (i.e., circadian) rhythms produced by these clock-genes are synchronized to the exact 24-h rhythms in the outer world by their sensitivity to (sun)light (Dibner et al., 2010). The synchronizing stimuli for peripheral clocks in non-SCN tissues are less clear, in addition to nervous and humoral signals from the SCN, behavioral signals such as body temperature, energy metabolism and (feeding) activity also likely play a role (Hastings et al., 1998; Dibner et al., 2010).

**Figure 1:**
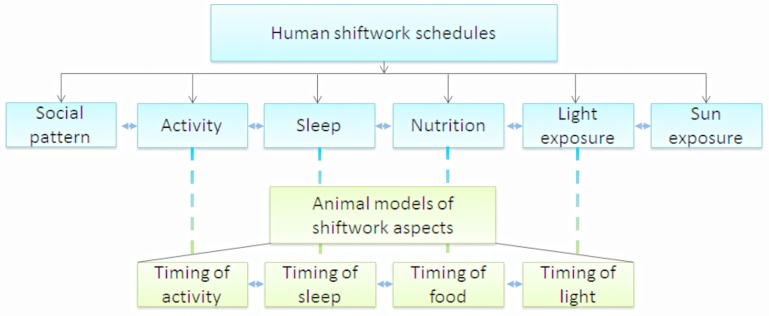
**Shiftwork can be disentangled into different aspects (blue blocks), for some of these aspects animal models have been developed (green blocks)**. Each of these aspects might contribute to health risks associated with shiftwork. However, all aspects strongly interact, making it difficult to separate the effects of each single aspect. In most animal studies only one of the aspects is manipulated, however, it is important to keep in mind that by manipulation of one aspect, other aspects might be affected as well due to this interaction.

In general, the working schedules of shiftworkers profoundly interfere with these normal daily rhythms (Puttonen et al., 2010; Fritschi et al., 2011). Shiftwork leads to a disruption of the circadian rhythms produced by the central and peripheral clocks by confronting them with opposing signals, i.e., light at night and food consumption and activity during the sleep period. Therefore, shiftwork is a challenge that contains many aspects, which might be related to the negative health effects (Figure 1): (1) social pattern: shiftwork affects social life due to working hours that conflict with working hours of social contacts; (2) activity: shiftwork affects the timing of people's activity and, as a consequence, possibly affects the amount of activity; (3) sleep: shiftwork affects timing of sleep and possibly duration and quality of sleep; (4) nutrition: shiftwork affects timing of food intake and possibly meal frequency and composition; (5) light exposure: shiftwork affects the timing of light exposure, with possibly different intensity and duration of exposure; (6) sun exposure: shiftwork might affect the duration of sun exposure and as a consequence vitamin D levels. Shiftwork comprises alterations at different levels of the circadian system that each have their own effect, but are interacting as well (Figure 1; Puttonen et al., 2010; Fritschi et al., 2011). For example, the altered timing of activity in shiftworkers may result in sleep disturbances (duration, quality, and timing), altered nutrition (composition, caloric intake and timing), changed lighting exposure conditions (duration, intensity, and timing), reduced sunlight exposure (possible effect on vitamin D levels) and disturbances in social life. Each of these aspects might, to a greater or lesser extent, contribute to negative health effects. For several aspects, animal models have been developed to examine the metabolic health effects upon manipulation of these aspects of shiftwork individually or in combination (Figure 1). To our knowledge, no animal models have so far been developed to study effects of “social life” and “sun exposure.”

The aim of the present review is to provide an overview of the available animal studies investigating the mechanism underlying the negative health consequences of shiftwork and their outcome. In addition, we discuss human relevancy of the available animal models for shiftwork to gain insight into animal to human translatability and aid future investigations in choosing optimal animal models. Next to animal models mimicking circadian disruptive shiftwork aspects, consequences of circadian disruption have also been studied in animals using genetic manipulation or SCN lesions. These animal models are not within the scope of the present review, since we do not consider them to represent human shiftwork.

To increase the animal to human translatability the focus of the present review is on animal models investigating the relationship between shiftwork and metabolic risk factors (Haffner, 1998; Carnethon et al., 2004; Esquirol et al., 2011) since these factors are easily translatable from humans to animals and vice versa. In addition, these factors can be measured almost non-invasively in humans and often appear before the full blown disease, allowing for shorter follow-up time and more time for preventive measures or intervention.

Studies were included in the review when they investigated metabolic parameters such as bodyweight, food intake, activity, glucose metabolism (including plasma glucose, insulin and glucagon levels, glucose tolerance, and glycogen levels), leptin levels, and lipid metabolism (including plasma cholesterol and triglyceride levels).

## Methods

### Search strategy

A literature search was performed to obtain an overview of the current scientific literature on studies using animal models for shiftwork to investigate the relationship between shiftwork and metabolic function. The search strategy was designed by an information specialist (RIVM) using the MESH-database of Pubmed, to include all MeSH terms and its synonyms and several electronic databases were used (Medline, Embase, BIOSIS Previews en SciSearch). In brief, the search strategy combined keywords related to shiftwork with keywords related to metabolic risk factors. Examples of key words for shiftwork: shift work^*^, shiftwork^*^, night work^*^, night shift^*^, rotating shift^*^, jet lag, working rhythm^*^, “irregular working hours,” time restricted, “constant light,” “continuous light,” “light at night,” biological clock^*^, body clock^*^, chronobiology^*^, circadian clock^*^. Examples of key words for metabolic risk factors: weight, body weight, weight change, metabolic syndrome, obesity, adiposity, glucose, glucose tolerance, lipid metabolism, energy metabolism, insulin, insulin sensitivity, hypertension, leptin. Only papers published after 1993 were included. For the complete search strategy see Supplementary Data.

The search resulted in 1550 publications, but only 44 were included as these met the following criteria:

1. Using animal models for shiftwork
2. Investigate effects on at least one of the following metabolic risk factors for disease (Haffner, 1998; Carnethon et al., 2004):

bodyweight and related measures (BMI, fat percentage) or glucose homeostasis: including plasma glucose levels, glucose tolerance, plasma insulin levels, insulin sensitivity or lipid homeostasis: including plasma levels of triglycerides, cholesterol, free fatty acids, HDL or LDL.

The search included papers in English, Dutch, French, and German. However, only papers in English fitted the above mentioned criteria. In addition to this search strategy, the present knowledge of the authors and references from included papers (“snowball method”) were used to include additional papers that fitted the above mentioned criteria (including papers published before 1993) or investigated parameters related to metabolic dysfunction.

### Categorization of studies

Included studies were divided in four different categories as presented in Figure 1: (1) Models using “timing of food intake,” (2) models using “timing of activity,” (3) models using “timing of sleep,” and (4) models using “timing of light.” For each study the outcome parameters were determined, which included the abovementioned metabolic risk factors for disease as well as circadian parameters (e.g., activity, cortisol) and gene expression. For these parameters, results are described in the text and summarized in the table for overview purposes. In the Tables 1–**5**, the left column holds the metabolically relevant parameters which were scored for and were most frequently measured in the studies. The *rat* and *mouse* columns represent the number of studies in which an effect of the manipulation (compared to the control condition) was found in this species against the number of studies in which it was measured. In the *total* column, results are divided in direction of effects and presented as the number of studies observing that direction is summarized. This was not done for gene expression. The most right column shows the studies in which the parameter was measured in this category of models. In the tables, studies are counted twice when multiple experiments are performed in one article, for instance when two types of mice were used. Occasionally, the effect of the manipulation was measured on the total level as well as the rhythm of a parameter within the same study. In this case, both effects are included in the *total* column.

**Table 1:** **Summary of animal studies in which timing of food intake was manipulated to mimic human shiftwork**.

**Food**	**Rat**	**Mouse**	**Total**	**Studies**
Bodyweight	1/3	6/8	+: 3/11 (27, 3%)	Arble et al., 2009; Bray et al., 2010, 2013; Salgado-Delgado et al., 2010a, 2013; Jang et al., 2012; Sherman et al., 2012; Yoon et al., 2012; Reznick et al., 2013; Oyama et al., 2014; Shamsi et al., 2014
			−: 4/11 (36, 4%)	
			o: 4/11 (36, 4%)	
Food intake total	1/3	4/8	+: 0/11 (0%)	Arble et al., 2009; Bray et al., 2010, 2013; Salgado-Delgado et al., 2010a, 2013; Jang et al., 2012; Sherman et al., 2012; Yoon et al., 2012; Reznick et al., 2013; Oyama et al., 2014; Shamsi et al., 2014
			−: 5/11 (45, 5%)	
			o: 6/11 (54, 5%)	
Activity total	1/2	2/5	+: 2/7 (28, 6%)	Arble et al., 2009; Bray et al., 2010, 2013; Salgado-Delgado et al., 2010a; Sherman et al., 2012; Yoon et al., 2012; Reznick et al., 2013; Shamsi et al., 2014
			−: 1/7 (14, 3%)	
			o: 4/7 (57, 1%)	
EE total	1/1	2/2	+: 0/3 (0%)	Arble et al., 2009; Bray et al., 2010, 2013; Reznick et al., 2013
			−: 3/3 (100%)	
			o: 0/3 (0%)	
RER	1/1	2/2	+: 1/3 (33, 3%)	Bray et al., 2010, 2013; Reznick et al., 2013
			−: 1/3 (33, 3%)	
			~: 1/3 (33, 3%)	
			o: 0/3 (0%)	
Adiposity	2/3	3/4	+: 3/7 (42, 8%)	Arble et al., 2009; Bray et al., 2010; Salgado-Delgado et al., 2010a, 2013; Sherman et al., 2012; Reznick et al., 2013; Shamsi et al., 2014
			−: 2/7 (28, 6%)	
			o: 2/7 (28, 6%)	
Glucose metabolism	2/3	4/6	+: 1/9 (11, 1%)	Bray et al., 2010, 2013; Salgado-Delgado et al., 2010a, 2013; Jang et al., 2012; Sherman et al., 2012; Yoon et al., 2012; Reznick et al., 2013; Shamsi et al., 2014
			−: 2/9 (22, 2%)	
			~: 3/9 (33, 3%)	
			o: 3/9 (33, 3%)	
Lipid metabolism	2/2	3/5	+: 1/7 (14, 3%)	Bray et al., 2010, 2013; Salgado-Delgado et al., 2010a; Sherman et al., 2012; Yoon et al., 2012; Reznick et al., 2013; Shamsi et al., 2014
			−: 2/7 (28, 6%)	
			~: 4/7 (57, 1%)	
			o: 2/7 (28, 6%)	
Corticosterone	2/2	2/3	+: 1/5 (20%)	Salgado-Delgado et al., 2010a; Sherman et al., 2012; Bray et al., 2013; Reznick et al., 2013; Shamsi et al., 2014
			−: 0/5 (0%)	
			~: 3/5 (60%)	
			o: 1/5 (20%)	
Melatonin				
Leptin	1/1	0/1	+: 1/2 (50%)	Bray et al., 2010; Sherman et al., 2012; Reznick et al., 2013
			−: 0/2 (0%)	
			~: 1/2 (50%)	
			o: 1/2 (50%)	
Ghrelin		1/1	+: 0/1 (0%)	Sherman et al., 2012
			−: 1/1 (100%)	
			~: 0/1 (0%)	
			o: 0/1 (0%)	
BP/Heart rate		2/2	+: 0/2 (0%)	Schroder et al., 2014
			−: 2/2 (100%)	
			~: 0/2 (0%)	
			o: 0/2 (0%)	
Gene expression	2/2	10/10	12/12 (100%)	Damiola et al., 2000; Jang et al., 2012; Sherman et al., 2012; Yoon et al., 2012; Bray et al., 2013; Reznick et al., 2013; Salgado-Delgado et al., 2013; Oyama et al., 2014; Shamsi et al., 2014

*For detailed description of the columns, see Methods section*.

*+, number of studies with increases; −, number of studies with decreases; ~, number of studies with altered rhythm; o, number of studies with no effect. For glucose metabolism a + indicates increased basal levels of glucose, HbA1c or insulin, increased HOMA index or decreased glucose tolerance*.

*EE, energy expenditure; RER, respiratory exchange ratio; BP, blood pressure*.

With these tables we aim to provide an overview of the metabolic parameters that are influenced per category and type of animals used. Results of effect on gene expression are described in the text and summarized in the table as “gene expression.” The present review was aimed at providing a narrative overview of available studies and their findings, due to heterogeneity studies were not assessed for quality.

## Results

Animal studies that model human shiftwork can be divided into four main categories. The first three categories are based on desynchronization of peripheral clocks from the central clock by the unnatural timing of food intake, sleep or activity. The remaining category consists of studies that manipulate the timing of light exposure, including alterations of duration (i.e., continuous light) and timing of light exposure. Some studies used a shiftwork model that combines multiple categories and those will be mentioned repeatedly in the different categories if appropriate. Below we discuss the main findings of studies using a shiftwork model within the categories where the models fit best.

### Category 1: models using “timing of food intake”

Shiftwork models using shifted and/or restricted timing of food availability are based on the knowledge that food intake is the most important Zeitgeber for peripheral clocks, in the same way as light is for the central clock. Shifting timing of food intake disrupts the orchestrated synchrony between peripheral and brain clocks, which might lead to metabolic problems as peripheral organs such as liver and muscle are essential for energy homeostasis. Shifting the timing of food intake is an interesting approach as metabolic disorders such as obesity are also associated with aberrant dietary habits (i.e., quantity, composition and frequency) and shiftworkers also have changed dietary habits. Moreover, more recently several studies have suggested that also the timing of food intake is crucial to maintain energy homeostasis (Gluck et al., 2011; Garaulet et al., 2013; Hibi et al., 2013; Garaulet and Gomez-Abellan, 2014; Wang et al., 2014) and shifting the timing of food intake is another characteristic feature of shiftworkers (Lowden et al., 2010). All in all making this a relevant model for shiftwork.

The first evidence for food as a strong entrainment signal for circadian physiology (metabolic and clock gene expression rhythms, hormone secretion rhythms) came from so-called restricted feeding studies. This type of studies usually restricts food availability to a short period (e.g., 2–4 h) during the light phase (which is the resting phase of nocturnal rodents) to study entrainment of peripheral clocks. Clearly these are important studies for chronobiology in general and still are performed frequently to look for and try to understand better the food entrainable oscillator. However, such restricted-feeding models are not an adequate reflection of human food intake behavior during shiftwork as they restrict the duration of food intake to a (very) short period and therefore were not included in this review.

Food restriction studies in which food availability is shifted or restricted to a certain phase of the day (i.e., a large part of or the complete light period or dark period) provide a more suitable approach to mimic human feeding behavior during shift work. One of the first studies with food availability restricted to either the 12-h light or 12-h dark phase was done by Damiola et al. and they showed a strongly disturbed circadian rhythm according to altered daily body temperature rhythms in mice that could eat only during the light (i.e., resting) phase. Several clock genes in liver adjusted their expression to the timing of food intake (Damiola et al., 2000). Although alterations in gene expression cannot be translated directly into functional changes, it does indicate that food has strong entraining properties even on a molecular level in mice. More recently, Bray et al. performed a short-term experiment and observed metabolic changes within the first 9 days after restricting food intake to the light or dark phase. Whole body energy metabolism was affected within 24 h of food restriction and this was visible in a 5 h phase advance of rhythm in energy expenditure, higher resting energy expenditure (RER) and increased caloric intake. Restricting food to the resting phase caused an increase of bodyweight and blunted plasma glucose and corticosterone rhythm, whereas triglyceride levels were not affected (Bray et al., 2013). A short term experiment by Oyama et al. focused on the effects of food timing on inflammatory response but additionally found reduced food intake and bodyweight in mice fed during the light phase (Oyama et al., 2014). Jang et al. performed the same restriction protocol but studied long-term effects (5–9 weeks). Surprisingly, they observed a protective effect of restricting food to the resting phase with lower bodyweight and food intake when compared to (chow or high-fat diet) *ad libitum* fed animals. Bodyweight did not differ from animals pair-fed during the active phase. Also, alterations in the expression of lipogenic, gluconeogenic and fatty acid oxidation-related genes in liver were found in feeding time-restricted animals (Jang et al., 2012). Shamsi et al. entrained mice to 16L:8D or 8L:16D photoperiods and restricted food availability to light- or dark phase. Neither photoperiod nor food timing affected bodyweight when compared to *ad libitum* feeding. Plasma insulin increased in light phase fed animals despite the photoperiod, whereas plasma glucose tended to be lower and triglycerides significantly decreased when feeding during light was compared to *ad libitum* or dark phase feeding. Rhythms in plasma glucose, insulin and triglyceride secretion shifted by light phase feeding when compared to dark phase feeding and some effects (mostly amplitude) changed over time (i.e., 7 vs. 35 days). Interestingly, long photoperiod caused light phase fed animals to increase glucose tolerance but decrease insulin tolerance compared to dark phase fed animals and *ad libitum* fed animals respectively. Gene expression of metabolic and clock genes in liver was altered by feeding during light phase in both amplitude and phase. Corticosterone rhythm was shifted by light phase feeding after 35 days but not after 7 days (Shamsi et al., 2014). Reznick et al. took a similar approach with a 3-week study performed with Wistar rats instead of mice. No effect was found on bodyweight gain or epididymal white adipose tissue, but animals fed during the resting phase decreased their food intake and total activity levels. Rats fed during the light period showed a 12-h shift in RER and dampening of activity and energy expenditure diurnal variation. The rhythm of plasma insulin altered with higher 24-h levels, corticosterone showed an additional peak and the rhythm of glycogen shifted to an opposite phase in liver but showed increased levels in muscle. Triglyceride levels in liver were reduced whereas muscle content was unaffected in animals fed during the light period. Expression of several other proteins and genes involved in energy metabolism and clock regulation in liver and muscle tissue showed phase changes or altered expression levels. In the same study this experimental design was used for a group of rats fed a high fat diet which aggravated many of the observed effects found in chow day-fed animals with additional disruption of leptin and NEFA (non-esterified fatty acids) levels (Reznick et al., 2013).

A series of studies performed with male Wistar rats used a forced activity protocol as a model for shiftwork (Salgado-Delgado et al., 2008, 2010a,b, 2011, 2013). The effects of forced activity will be described below (see category 2), but the non-working “control” groups of these studies are relevant for the timing of food category. When food was restricted to the resting phase, i.e., chow only available from ZT0 to ZT12, rats displayed a dampening of their core body temperature rhythm, an additional peak in the plasma corticosterone rhythm, and a shift in triglyceride secretion, but no differences were observed for the plasma glucose and activity rhythm or total activity. Total food intake remained the same but bodyweight and peritoneal fat accumulation were increased when compared to *ad libitum* or night fed (food available from ZT12 to ZT24) animals (Salgado-Delgado et al., 2010a). The observed accumulation of abdominal fat was reproduced by the same group in another study where a decreased glucose tolerance in rats fed during the resting phase was observed, in addition to alterations or dampened rhythms in liver clock and metabolic gene expression (Salgado-Delgado et al., 2013).

A couple of other groups used comparable food availability approaches in mice on normal chow diet, however, shorter food restriction periods were used than 12 h during the light period. Yoon et al. enforced a 6-h advance (ZT6-11) or delay (ZT18-23) in food availability for 9 weeks and observed that body temperature, locomotor activity and triglyceride secretion strongly depend on food timing. Cholesterol and HDL levels were moderately increased in both advance and delay groups when compared to *ad libitum* fed animals, and food intake was reduced in the food time advanced group compared to food time delay group and ad-lib controls. Fasting glucose levels increased and poor responses to insulin tolerance test intensified over time in daytime fed animals (advance group) (Yoon et al., 2012). Sherman and colleagues restricted food intake to the light phase, ZT4-8 without caloric restriction, for 18 weeks and performed this with both high- and low-fat (chow) diets. In both diets, time restriction was protective for bodyweight gain, high plasma leptin, insulin, HDL and cholesterol levels. Also the increased epididymal fat observed in *ad libitum* fed animals was diminished in the food time-restricted groups. In low fat diet fed animals triglyceride levels were reduced but corticosterone and adiponectin were increased, as compared to the *ad libitum* low and high-fat and restricted high-fat animals. High-fat diet fed animals also showed improved TNF-alpha and HOMA-IR levels and increased activity levels when food was restricted to the light period, as compared to *ad libitum* fed animals, but were less active than animals on a low-fat diet (Sherman et al., 2012). Schroder et al. focused on the effects on heart rhythm and observed that when food was provided from ZT2-9 only, this negatively affected several aspects of heart rhythm aspects in both wild type and genetically sensitive mice (Schroder et al., 2014).

Most studies mentioned above were performed with normal chow diet which is low on fat derived content. However, many diet-induced obesity studies use high-fat diet *ad libitum* feeding on which animals will develop obesity, diabetes and metabolic syndrome (Zaragoza and Felber, 1970). For circadian studies it is important to know that feeding rodents a high-fat diet induces a dampening of the amplitude of daily activity and feeding rhythms, and also metabolic markers, hypothalamic neuropeptides and peripherally expressed factors involved in lipid metabolism are affected (Kohsaka et al., 2007). This suggests an interaction between energy metabolism and circadian rhythm control. Some groups, however, combined the restricted-feeding paradigm with a high-fat diet. Restriction of food to one phase of the day may re-induce the entrainment lost on high-fat *ad libitum* feeding. Arble and colleagues made an early attempt and fed mice a high-fat diet solely during the light-phase and observed a significant increase in bodyweight compared to animals fed during the dark phase (Arble et al., 2009). Bray et al. used four different feeding schedules to study in more detail which phase of the L/D-cycle is most detrimental when ingesting a high-fat diet for 12 weeks. They observed higher bodyweight gain and adiposity, decreased glucose tolerance next to high insulin, leptin and triglyceride levels in mice consuming their high-fat meal at the end of the active phase instead of at the beginning. Interestingly, when fat was only available in the light phase no metabolic changes were observed with the exception of slightly decreased energy expenditure and oxygen consumption when compared to animals with fat available in the dark phase (Bray et al., 2010). Several studies experimented with restriction of a nutrient component to a certain phase of the day for the effects on obesity. For instance, providing the fat component or sugar component of a free-choice high-fat-high-sugar diet only during the light phase affects RER, energy expenditure and bodyweight (La Fleur et al., 2014; Oosterman et al., 2014). A slightly different approach was taken by Senador et al. by offering mice a 10% fructose solution additional to their normal chow and water diet. Fructose was either available for 24 h, available for 12 h during light phase, available for 12 h during dark phase or not available. Increased bodyweight, higher fasting glucose levels but decreased plasma triglycerides were observed in both groups with 12 h fructose availability. Fructose restriction to light phase additionally caused glucose intolerance and an attenuated amplitude of blood pressure rhythms. *Ad libitum* availability of fructose only caused glucose intolerance when compared to control animals without fructose (Senador et al., 2012). Another type of timed food restriction is done by dividing food intake into 4 or 6 meals equally divided over the L/D-cycle. For instance, Yamajuku et al. delivered a high cholesterol diet to rats in a 4-meal schedule (every 6 h) without reduction of caloric intake. Those animals developed hypercholesterolemia after 7 days on the protocol and furthermore showed disruption in liver gene expression (Yamajuku et al., 2009). These studies are probably highly relevant as shiftwork models. However, until now it is unclear how shift workers exactly change their dietary habits in timing, composition, frequency and size of meals, making it hard to decide at present what are the best models.

In contrast to studies that restrict food availability to the light phase, Hatori et al. showed that restricting a high-fat diet to the natural main feeding phase (ZT13-21) improved glucose tolerance, insulin sensitivity, adiposity, serum cholesterol levels and leptin levels after fasting or glucose administration, next to prevention of increased liver size and unsaturated fatty acids levels, compared to animals fed *ad libitum* (Hatori et al., 2012).

#### Summary “timing of food intake” models

Animal models using a restriction of the timing of food intake affect bodyweight in 64% of the studies, but effects went in different directions with 3 out of 7 studies showing an increase of bodyweight whereas the four other studies found a decrease in bodyweight after timed food intake. Restricting food to the light phase resulted in increased bodyweight compared to animals with food restricted to the dark phase (Arble et al., 2009; Bray et al., 2013) and when compared to *ad libitum* fed (Salgado-Delgado et al., 2010a). However, others observed reduced bodyweight after food restriction to the light phase compared to dark fed animals (Yoon et al., 2012; Oyama et al., 2014) or *ad libitum* fed animals (Sherman et al., 2012; Yoon et al., 2012). Interestingly, in many of these studies food intake was reduced as well. Furthermore, some differences between mouse and rat studies are observed. For example, for bodyweight, mice studies show a significant effect in 6 out of 8 studies (75%), whereas in rats only in 1 out of the 3 studies (33%) a significant effect was observed. Total food intake and glucose metabolism parameters were measured frequently and were affected in 45 and 67% of the studies, respectively. For a complete overview of all parameters see Table 1. Together, these results indicate that changing the timing of food intake is effective at influencing several metabolic parameters, although for some parameters results are not consistent. Considering the great variety in types of modulations used it is not possible to pinpoint this to one aspect of the models used.

### Category 2: models using “timing of activity”

“Work” is a difficult concept to model in animal studies and therefore only a very limited number of studies are available using an actual physical shiftwork protocol. Obviously shiftwork requires shifting of phases of sleep and arousal leading, at least partly, to awakening during the usual sleep period and sleep during the usual active period of the day. The few available physical shiftwork models used forced activity by housing animals in slowly rotating wheels. These housing conditions force the animals to be active and prevent them to fall asleep, although the animals can lie down and eat. Salgado-Delgado et al. mimicked human shiftwork protocols by enforcing the working conditions for 8 h per day, either during the active phase or the sleeping phase, 5 days a week for 5 weeks. In addition, they varied the availability of food but always used normal chow diets. A general observation in the groups of animals working during their resting phase was that the animals lost their nocturnal urge to eat and voluntarily consumed their food mostly during their working hours and thereby during their normal resting (i.e., light) phase. Forced activity during the resting phase induced increased bodyweight and abdominal fat, impaired glucose tolerance, altered plasma triglyceride diurnal variation, dampened daily glucose variation and introduced a secondary peak in the corticosterone rhythm. These effects could be prevented when food availability was restricted to the normal active (i.e., dark) phase (Salgado-Delgado et al., 2008, 2010a,b, 2011, 2013).

An early study by Tsai et al., used an extensive design with rats exposed to changes of light schedules (twice a week), forced activity paradigms and combinations of those. They observed higher bodyweight in the first 2 months, but not in the third month, in animals that were subjected to a “shiftwork” schedule of forced activity (i.e., Tue-Thu work from ZT0-ZT12, Fri-Sun work from ZT12-ZT24, Monday free) combined with changes in light schedule which was in synch (i.e., 12 h shift of L:D cycle twice a week) with the work schedule. Animals exposed to forced activity schedules had lower bodyweight and lower levels of cholesterol than animals that only underwent shift of light/dark cycle, indicating that forced activity can reverse some effects on metabolism induced by the regular L/D shifts. Some early effects on bodyweight and food intake disappeared after 2 months on the protocol, whereas other effects were only found after a few months on this protocol (Tsai and Tsai, 2007).

A study by Leenaars et al. using forced activity in rats did not focus on metabolic parameters, but did observe a decrease in total activity levels in animals that had to work either during their active or during their resting phase compared to freely active animals. Animals working shiftwork (i.e., work during the normal resting phase) showed reduced bodyweight gain compared to non-working controls (Leenaars et al., 2012).

A study by Hsieh et al. used 5-week forced activity in rats and closely studied alterations in activity patterns. The animals in this study are from the same study as Salgado-Delgado et al. (2008) but reported more specifically on locomotor activity. A decrease in mean activity levels was observed in shiftwork animals (i.e., working ZT2-10) during weekdays from week 3 onwards and during weekend days from week 1 onwards, but not in animals working during their active phase (i.e., working from ZT14-22). Shiftwork animals showed decreased amplitude of the activity rhythm on weekend days and a differently shaped rhythm of 24-h activity on weekdays. Shiftwork animals showed different activity responses to lights on and off when compared to non-shiftworking rats (Hsieh et al., 2014). The observed disruption of normal activity patterns was reported as an indication for circadian disruption as similar changes have been observed in SCN lesioned animals.

#### Summary “timing of activity” models

Models using an altered timing of activity have only been carried out with rats and therefore no numbers on mouse studies are available. The limited number of studies and contributing research groups with this paradigm resulted in high numbers of affected studies on all parameters. As shown in Table 2, all parameters showed a 100% effectiveness of the different studies, except for bodyweight, total food and total activity levels intake which were affected in 5, 2, and 4 out of 6 studies, respectively. These results suggest that “timing of activity” has metabolic effects, although considering the limited number of studies and research groups these results should be interpreted with some caution.

**Table 2:** **Summary of animal studies in which timing of activity was manipulated to mimic human shiftwork**.

**Activity**	**Rat**	**Mouse**	**Total**	**Studies**
Bodyweight	5/6		+: 3/6 (50%)	Murphy, 2003; Tsai and Tsai, 2007; Salgado-Delgado et al., 2008, 2010a, 2013; Leenaars et al., 2012
			−: 2/6 (33, 3%)	
			o: 1/6 (16, 7%)	
Food intake total	2/6		+: 1/6 (16, 7%)	Murphy, 2003; Tsai and Tsai, 2007; Salgado-Delgado et al., 2008, 2010a,b, 2013
			−: 1/6 (16, 7%)	
			o: 4/6 (66, 7%)	
Activity total	4/6		+: 0/6 (0%)	Salgado-Delgado et al., 2008, 2010a,b, 2013; Leenaars et al., 2012; Hsieh et al., 2014
			−: 4/6 (66, 7%)	
			o: 2/6 (33, 3%)	
EE total				
RER				
Adiposity	2/2		+: 2/2 (100%)	Salgado-Delgado et al., 2010a, 2013
			−: 0/2 (0%)	
			o: 0/2 (0%)	
Glucose metabolism	3/3		+: 1/3 (33, 3%)	Salgado-Delgado et al., 2008, 2010a, 2013
			−: 2/3 (66, 7%)	
			~: 0/3 (0%)	
			o: 0/3 (0%)	
Lipid metabolism	2/2		+: 0/2 (0%)	Salgado-Delgado et al., 2008, 2010a
			−: 0/2 (0%)	
			~: 2/2 (100%)	
			o: 0/2 (0%)	
Corticosterone	2/2		+: 0/2 (0%)	Salgado-Delgado et al., 2008, 2010a
			−: 0/2 (0%)	
			~: 2/2 (100%)	
			o: 0/2 (0%)	
Melatonin				
Leptin				
Ghrelin				
BP/Heart rate				
Gene expression	1/1		1/1 (100%)	Salgado-Delgado et al., 2013

*For detailed description of the columns, see Methods section*.

*+, number of studies with increases; −, number of studies with decreases; ~, number of studies with altered rhythm; o, number of studies with no effect. For glucose metabolism a + indicates increased basal levels of glucose, HbA1c or insulin, increased HOMA index or decreased glucose tolerance*.

*EE, energy expenditure; RER, respiratory exchange ratio; BP, blood pressure*.

### Category 3: models using “timing of sleep”

Alterations in timing of activity are directly related to alterations in timing and duration of sleep. However, changes in the timing of sleep are also separately used as a model for shiftwork. These studies are different from the previously described activity models as their first target is to disturb or shorten sleep and affect the timing of sleep, but not necessarily alter activity levels or food intake. However, undoubtedly changes in sleep behavior will also affect activity and feeding patterns. Interestingly, (chronic) sleep restriction is associated with metabolic disorders in both animals and humans (Gangwisch, 2009; Killick et al., 2012). We came across a diversity of methods used to disrupt the normal sleep/wake pattern, including sleep disruption, sleep restriction, sleep fragmentation, sleep perturbation or sleep deprivation. Some of those might be other designations of the same intentions, such as sleep fragmentation and perturbation. These models either use shifting the timing of the normal sleep phase along the light-dark cycle, perturbing sleep in the normal phase, reducing the total number of sleep hours or completely withholding sleep. Most of these sleep studies focused on the effects of sleep perturbation on sleep parameters (such as percentage REM and NREM sleep, EEG recordings), behavioral changes or other non-metabolic factors. In this review only studies using a shift in the timing of sleep to the dark phase and studies using total sleep deprivation for a few hours were included as those were considered most relevant for shiftwork models.

Methods to perturb sleep are diverse and forced activity, as mentioned before, is one of them. Another method involves gentle handling for a few hours, by touching the animal every time it tries to fall asleep. Short term effects of sleep restriction during the first 6 h of the normal sleep phase (ZT0-ZT6) were described in two studies. Barclay et al. observed moderate alterations in the timing of food intake (in the direction of light phase feeding) and locomotor activity (increased levels during light and decreased levels during dark phase), next to major disruptions in liver transcriptome rhythms enriched for lipid and glucose metabolism pathways after 2 weeks of sleep restriction. A decreased response in the pyruvate test, a dampening of the daily rhythms in plasma glycerol, plasma triglyceride, plasma corticosterone and hepatic glycogen levels, and disrupted expression of several clock genes were all rescued by restricting food intake to the dark phase in the sleep restricted groups (Barclay et al., 2012).

Another study (Husse et al., 2012) used the same method but subjected the animals to sleep restriction for only five consecutive days followed by a recovery week during which several parameters were measured. Sleep restriction led to increased food intake despite increased leptin levels, together these changes are indicative for leptin resistance. Blood metabolites such as glucose and triglycerides were increased, but levels improved again during the recovery week. However, a trend toward higher bodyweight gain was observed during the recovery week suggesting long term effects even when the period of sleep restriction has terminated. Analysis of white adipose tissue transcriptome showed that sleep restriction affects many genes involved in lipid metabolism, including increased fatty acid synthesis and triglyceride production and storage.

A series of sleep restriction studies (Barf et al., 2010, 2012a,b) have been done using sleep restriction protocols of different severities: sleep restriction for 20 h each day (sleep ZT0-4; awake ZT4-24) or sleep disruption (14 h sleep—10 h awake in four 2–3 h episodes). Sleep disturbance and sleep restriction both led to decreased bodyweight when compared to home cage control animals, although food intake was equal and the slight increase in locomotor activity is unlikely to explain the bodyweight differences. Animals exposed to sleep disruption or sleep restriction showed decreased baseline plasma glucose and insulin levels, decreased glucose tolerance and an attenuated insulin response to the glucose infusion within 8 days. A five day recovery period attenuated the sleep restriction-induced decrease of plasma leptin, insulin and glucose levels although bodyweight gain did not recover. Corticosterone levels and food intake were not affected (Barf et al., 2012b). When the same protocol was performed for 4 weeks, but with a work-weekend-schedule (5 work days, 2 non-work days) to resemble human shiftwork conditions, the same authors observed an increase of food intake after the first weekend, possibly to compensate for increased energy expenditure. Bodyweight gain increased during weekends in sleep restricted animals and plasma leptin and insulin levels were decreased when measured during working weeks 1 and 4. Rest during weekend days induced recovery of plasma leptin and insulin levels (Barf et al., 2012a).

#### Summary “timing of sleep” models

The number of studies using perturbation of sleep as a model for shiftwork with focus on metabolic parameters is limited and therefore it is not yet completely clear if this type of manipulation influences metabolic functioning. Glucose metabolism appears to be affected often (in 5 out of 5 studies), whereas bodyweight (3/5) and food intake (2/4) were not always affected by sleep perturbation. For a complete overview of all parameters see Table 3.

**Table 3:** **Summary of animal studies in which timing of sleep was manipulated to mimic human shiftwork**.

**Sleep**	**Rat**	**Mouse**	**Total**	**Studies**
Bodyweight	3/3	0/2	+: 1/5 (20%)	Barf et al., 2010, 2012a,b; Barclay et al., 2012; Husse et al., 2012
			−: 3/5 (60%)	
			o: 2/5 (20%)	
Food intake total	1/2	1/2	+: 2/4 (50%)	Barf et al., 2010, 2012a; Barclay et al., 2012; Husse et al., 2012
			−: 0/4 (0%)	
			o: 2/4 (50%)	
Activity total	0/1	0/2	+: 0/3 (0%)	Barclay et al., 2012; Barf et al., 2012a; Husse et al., 2012
			−: 0/3 (0%)	
			o: 3/3 (100%)	
EE total	0/1		+: 0/1 (0%)	Barf et al., 2012a
			−: 0/1 (0%)	
			o: 1/1 (100%)	
RER				
Adiposity				
Glucose metabolism	3/3	2/2	+: 3/5 (60%)	Barf et al., 2010, 2012a,b; Barclay et al., 2012; Husse et al., 2012
			−: 2/5 (40%)	
			~: 0/5 (0%)	
			o: 0/5 (0%)	
Lipid metabolism		2/2	+: 2/2 (100%)	Barclay et al., 2012; Husse et al., 2012
			−: 1/2 (50%)	
			~: 0/2 (0%)	
			o: 0/2 (0%)	
Corticosterone	1/2	1/2	+: 2/4 (50%)	Barclay et al., 2012; Barf et al., 2012a,b; Husse et al., 2012
			−: 0/4 (0%)	
			~: 0/4 (0%)	
			o: 2/4 (50%)	
Melatonin				
Leptin	2/2	2/2	+: 1/4 (25%)	Barclay et al., 2012; Barf et al., 2012a,b; Husse et al., 2012
			−: 3/4 (75%)	
			~: 0/4 (0%)	
			o: 0/4 (0%)	
Ghrelin				
BP/heart rate				
Gene expression		2/2	2/2 (100%)	Barclay et al., 2012; Husse et al., 2012

*For detailed description of the columns, see Methods section*.

*+, number of studies with increases; −, number of studies with decreases; ~, number of studies with altered rhythm; o, number of studies with no effect. For glucose metabolism a + indicates increased basal levels of glucose, HbA1c or insulin, increased HOMA index or decreased glucose tolerance*.

*EE, energy expenditure; RER, respiratory exchange ratio; BP, blood pressure*.

### Category 4: models using “timing of light exposure”

In literature, several “timing of light exposure” models have been reported. These models all use timing of light as a means to disturb the circadian system and as such their main influence is on the SCN, in contrast to the previous 3 categories of models described. One type of these models uses continuous light exposure, i.e., light is present 24 h per day. The continuous light models can be further subdivided in models using constant light (similar amounts of light exposure during 24 h of the day) and models using dim light at night (together with bright light during the day). In addition to models using continuous light, models using changes in light/dark schedules have been used. These models can be further subdivided into models using alterations in period length (e.g., light-dark periods shorter or longer than 24 h) and models using repeated shifts of the light/dark schedule. Studies investigating these types of exposure in relation to metabolic health effects are discussed below per type of model. For overview purposes, models using continuous light are presented together in Table 4 and models using changes in light/dark schedules are presented together in Table 5.

**Table 4:** **Summary of animal studies in which continuous light (LL) or dimlight (LDim) at night exposure was used to mimic human shiftwork**.

**LL/LDim**	**RAT**	**Mouse**	**Total**	**Studies**
Bodyweight	1/5	5/6	+: 6/11 (54, 5%)	Natelson et al., 1993; Dauchy et al., 2010; Fonken et al., 2010; Gale et al., 2011; Coomans et al., 2013a; Aubrecht et al., 2014; Borniger et al., 2014
			−: 0/11 (0%)	
			o: 5/11 (45, 4%)	
Food intake total	0/2	2/7	+: 1/9 (11, 1%)	Dauchy et al., 2010; Fonken et al., 2010; Coomans et al., 2013a; Shi et al., 2013; Aubrecht et al., 2014; Borniger et al., 2014
			−: 1/9 (11, 1%)	
			o: 7/9 (77, 7%)	
Activity total		0/5	+: 0/5 (0%)	Fonken et al., 2010; Shi et al., 2013; Aubrecht et al., 2014; Borniger et al., 2014
			−: 0/5 (0%)	
			o: 5/5 (100%)	
EE total		2/2	+: 0/2 (0%)	Coomans et al., 2013a; Borniger et al., 2014
			−: 2/2 (100%)	
			o: 0/2 (0%)	
RER		2/2	+: 2/2 (100%)	Coomans et al., 2013a; Borniger et al., 2014
			−: 0/2 (0%)	
			o: 0/2 (0%)	
Adiposity		1/1	+: 1/1 (100%)	Shi et al., 2013
			−: 0/1 (0%)	
			o: 0/1 (0%)	
Glucose metabolism	3/4	3/3	+: 4/7 (57, 1%)	Dauchy et al., 2010; Fonken et al., 2010; Gale et al., 2011; Coomans et al., 2013a
			−: 0/7 (0%)	
			~: 2/7 (28, 6%)	
			o: 1/7 (14, 3%)	
Lipid metabolism	1/2		+: 0/2 (0%)	Dauchy et al., 2010
			−: 0/2 (0%)	
			~: 1/2 (50%)	
			o: 1/2 (50%)	
Corticosterone	2/2		+: 1/2 (50%)	Dauchy et al., 2010
			−: 0/2 (0%)	
			~: 2/2 (100%)	
			o: 0/2 (0%)	
Melatonin	4/4		+: 0/4 (0%)	Dauchy et al., 2010; Gale et al., 2011
			−: 2/4 (50%)	
			~: 2/4 (50%)	
			o: 0/4 (0%)	
Leptin				
Ghrelin				
BP/Heart rate				
Gene expression				

*For detailed description of the columns, see Methods section*.

*+, number of studies with increases; −, number of studies with decreases; ~, number of studies with altered rhythm; o, number of studies with no effect. For glucose metabolism a + indicates increased basal levels of glucose, HbA1c or insulin, increased HOMA index or decreased glucose tolerance*.

*EE, energy expenditure; RER, respiratory exchange ratio; BP, blood pressure*.

**Table 5:** **Summary of animal studies in which exposure to L/D shifts was used to mimic human shiftwork**.

**L/D shifts**	**Rat**	**Mouse**	**Total**	**Studies**
Bodyweight	2/5	3/4	+: 4/9 (44, 4%)	Vilaplana et al., 1995; Tsai et al., 2005; Bartol-Munier et al., 2006; Oishi, 2009; Gale et al., 2011; Karatsoreos et al., 2011; Oishi and Itoh, 2013; Voigt et al., 2014
			−: 1/9 (11, 1%)	
			o: 4/9 (44, 4%)	
Food intake total	2/3	1/3	+: 2/6 (33, 3%)	Vilaplana et al., 1995; Tsai et al., 2005; Bartol-Munier et al., 2006; Oishi, 2009; Karatsoreos et al., 2011; Oishi and Itoh, 2013
			−: 1/6 (16, 7%)	
			o: 3/6 (50%)	
Activity total	2/2		+: 0/2 (0%)	Tsai et al., 2005; Bartol-Munier et al., 2006
			−: 2/2 (100%)	
			o: 0/2 (0%)	
EE total				
RER				
Adiposity				
Glucose metabolism	2/3	3/3	+: 4/6 (66, 7%)	Bartol-Munier et al., 2006; Oishi, 2009; Gale et al., 2011; Karatsoreos et al., 2011; Oishi and Itoh, 2013
			−: 1/6 (16, 7%)	
			~: 0/6 (0%)	
			o: 1/6 (16, 7%)	
Lipid metabolism	0/1	1/2	+: 1/3 (33, 3%)	Bartol-Munier et al., 2006; Oishi, 2009; Oishi and Itoh, 2013
			−: 0/3 (0%)	
			~: 0/3 (0%)	
			o: 2/3 (66, 7%)	
Corticosterone				
Melatonin	2/2		+: 0/2 (0%)	Gale et al., 2011
			−: 0/2 (0%)	
			~: 2/2 (100%)	
			o: 0/2 (0%)	
Leptin		1/1	+: 1/1 (100%)	Karatsoreos et al., 2011
			−: 0/1 (0%)	
			~: 0/1 (0%)	
			o: 0/1 (0%)	
Ghrelin				
BP/Heart rate	1/1		+: 0/1 (0%)	Tsai et al., 2005
			−: 0/1 (0%)	
			~: 1/1 (100%)	
			o: 0/1 (0%)	
Gene expression		1/1	1/1 (100%)	Oishi and Itoh, 2013

*For detailed description of the columns, see Methods section*.

*+, number of studies with increases; −, number of studies with decreases; ~, number of studies with altered rhythm; o, number of studies with no effect. For glucose metabolism a + indicates increased basal levels of glucose, HbA1c or insulin, increased HOMA index or decreased glucose tolerance*.

*EE, energy expenditure; RER, respiratory exchange ratio; BP, blood pressure*.

#### Continuous light exposure

Exposure to constant bright light conditions (LL) has been shown to abolish/diminish the circadian rhythmicity of many laboratory animals. This has, for example, been shown for locomotor activity (Depres-Brummer et al., 1995; Gale et al., 2011), melatonin (Wideman and Murphy, 2009; Gale et al., 2011), food intake (Coomans et al., 2013b), and SCN neurons (Coomans et al., 2013b). Hence, constant bright light conditions may be used to severely disrupt circadian rhythms. Since shiftworkers will be exposed to light during most of the day these models might be relevant for shiftwork modeling, although the intensity of all-day light may differ from real-life light exposures.

Disruption of circadian rhythms by constant bright light exposure has also been reported to affect metabolic function. Exposure to continuous light (150–180 lux; 4–8 weeks) led to increased bodyweight gain in two studies in mice (Fonken et al., 2010; Coomans et al., 2013a). This phenotype was apparent in mice fed normal chow (Fonken et al., 2010; Coomans et al., 2013a) as well as a high-fat diet (Coomans et al., 2013a). In addition, a third study reported increased fat mass after exposure to continuous light (Shi et al., 2013). Total food intake was unaltered in these studies, but more food was consumed during the subjective day, indicating changes in the timing of food intake (Fonken et al., 2010; Coomans et al., 2013a). The effect of continuous light exposure on total activity levels is less clear: unaltered activity levels (Fonken et al., 2010), reduced energy expenditure (Coomans et al., 2013a) and a non-significant trend toward a decrease in activity levels (Shi et al., 2013) have been reported. Apart from changes in bodyweight and fat mass, continuous light affected other metabolic parameters, such as increased RER, reduced glucose tolerance and altered rhythmicity of insulin sensitivity (Fonken et al., 2010; Coomans et al., 2013a). However, two studies using relative short-term exposure (6–10 weeks) to continuous bright light in Sprague Dawley rats reported no changes in bodyweight (Dauchy et al., 2010; Gale et al., 2011), indicating a possible difference between rats and mice in this model. Interestingly, long term exposure to bright light for 35 weeks did enhance bodyweight in Rapp-Dahl rats (model for hypertension). In addition, increased systolic blood pressure was observed (Natelson et al., 1993). Short-term exposure to continuous bright light did increase glucose levels and alter the rhythmicity of lipids in the study by Dauchy et al. (2010). In contrast, in the study by Gale et al. changes in glucose metabolism (increased glucose levels and decreased glucose- and arginine- stimulated insulin secretion) were only observed in diabetes-prone HIP rats, but not in wild-type Sprague Dawley rats (Gale et al., 2011). Considering these contradicting results, the differences in duration and strains of rats used, as well as the limited number of studies no firm conclusions can be drawn regarding the effects of continuous bright light exposure on metabolic function in rats. On the other hand, in mice results are more consistent and indicate that disruption of circadian rhythms by continuous bright light exposure increases bodyweight and alters glucose metabolism. This is associated with an altered timing of food intake, but not with change in total amount of food consumed over 24 h (Table 4).

#### Dim light at night

Dim light at night (LDim) also affects circadian rhythmicity, but seems less disruptive for circadian rhythms compared to constant bright light. In respect to human circadian disruptions caused by shiftwork, models using dim light might be more relevant, since human shiftworkers will also experience alterations in the level of light exposure during a day, i.e., dim light at work in the office and bright light when commuting. In addition, models using dim light at night are relevant for studying the possible health consequences of light contamination at home, i.e., evening and nocturnal light is present in an increasing amount in our western society.

In contrast to constant bright light exposure, with dim light exposure at night circadian rhythms remain largely intact. This has for example been observed in rhythms of locomotor activity (Fonken et al., 2010) and corticosterone (Dauchy et al., 2010). Metabolic parameters including plasma levels of glucose and fatty acids also remain intact. Interestingly, dim light at night did affect the circadian pattern of food intake with relatively more food being consumed during the rest phase (Fonken et al., 2010). However, when the brightness of dim light exceeds a certain limit circadian rhythmicity of melatonin and corticosterone will be affected (Dauchy et al., 2010).

In three mice studies, exposure to dim light at night (for 2 and 6 weeks) increased bodyweight (Fonken et al., 2010; Aubrecht et al., 2014; Borniger et al., 2014). This was observed in male (Fonken et al., 2010; Borniger et al., 2014) and female mice (Aubrecht et al., 2014). In female mice, dim light at night resulted in decreased food intake after 4 weeks (Aubrecht et al., 2014). In addition, in dim light exposed animals increases in fat mass and reduced glucose tolerance were observed (Fonken et al., 2010), as well as changes in energy expenditure and RER (reduced whole body expenditure and increased carbohydrate over fat oxidation) (Borniger et al., 2014). In contrast, bodyweight was not affected in a rat study using exposure to dim light at night for 6 weeks (Dauchy et al., 2010). In this study, different intensities of dim light at night were used (0.02–0.08 μW/cm^2^ dim light). The highest intensity of dim light at night disrupted circadian rhythms of plasma corticosterone, melatonin and glucose, but bodyweight was not affected during the 6 weeks of exposure.

Considering the still limited number of studies using dim light, firm conclusions are not possible yet. However, it appears that similar to continuous bright light exposure dim light at night affects bodyweight in mice, but not in rats. The currently available studies suggest that glucose metabolism is affected in mice as well as in rats by dim light at night. Interestingly, the study by Fonken et al., reported that the increases in bodyweight gain and fat mass by dim light at night can be prevented by restricting food access to the dark phase (Fonken et al., 2010). These results suggest an important role for altered timing of food intake in the effects of continuous light on bodyweight, although none of these three dimlight studies clearly quantified the circadian changes in food intake.

#### Summary “constant light” models

Models using constant light seem to affect bodyweight in 55% of the studies (Table 4), all increases in bodyweight. However, there is a clear difference between rat and mice studies, with 1 out of 5 rat studies reporting effects on bodyweight and 5 out of 6 mice studies. Total food intake is not affected in most studies (only affected in 2 out of 9 studies), whereas glucose metabolism is affected in a majority of studies (6/7, 86%). Interestingly, the clear difference observed between rats and mice in the effects of constant light on bodyweight is not that pronounced for glucose metabolism. Thus, these results indicate that models using constant light exposure influence glucose metabolism in both species, while bodyweight is mainly affected in mice. For the other parameters only a limited number of studies are available making firm conclusions difficult. For a complete overview of all parameters see Table 4.

#### Changes in light/dark schedules—period length

Under normal conditions, one cycle of light and darkness on the planet earth matches exactly 24 h. Exposure to altered period lengths (<23 h or > than 25 h) usually requires a constant re-entrainment of the circadian system and experiments using such protocols have therefore been used to investigate the effects of circadian disruption. On the other hand, when very short period lengths are used entrainment is not possible, which will result in either free-running rhythms or an abolishment of circadian rhythms. Shorter period lengths have been reported to cause alterations in several circadian parameters, such as locomotor activity (Oishi, 2009; Oishi and Itoh, 2013), drinking pattern (Oishi, 2009) and body temperature (Karatsoreos et al., 2011).

Altered circadian rhythms due to an aberrant period length have been implicated in metabolic disturbances as well. For example, increases in bodyweight have been observed in mice and rats exposed to short period lengths of 6–23 h for 9–10 weeks (Vilaplana et al., 1995; Oishi, 2009; Karatsoreos et al., 2011; Oishi and Itoh, 2013). In addition, changes in glucose homeostasis (increased glucose levels and glucose intolerance), lipid homeostasis (increased cholesterol levels) and expression of liver genes related to glucose metabolism have been reported in one of these models (Oishi and Itoh, 2013). However, human relevancy of these models is poor since period length remains unaltered during shiftwork. Of course, partial shifts in light exposure might occur during shiftwork where light is present during working hours and is avoided during subsequent sleeping hours, but therefore models using shifts in light exposure are more relevant to the human situation than changes in period length.

#### Changes in light/dark schedules—shifts

Shifts in light exposure require re-entrainment of the circadian system causing (temporary) disturbance of circadian rhythms. Repeated phase shifts have been investigated using numerous schedules which differ in shift size (1–12 h), frequency (every day—once a week), duration (acute effects—chronic effects), and direction (forwards or backwards), resulting in very heterogeneous study results. For example, a 6 h forward shift every 3 days for 10 weeks abolished locomotor and melatonin rhythmicity (Gale et al., 2011), whereas rhythmicity in locomotor activity and body temperature remained but was disturbed (lengthened period and reduced amplitude) after an 8 h forward shift every 2 days for 10 days (Filipski et al., 2004).

Circadian disruption by shifts in light exposure has also been investigated in relation to metabolic function. To our knowledge, four studies using this type of model have been published. A study by Tsai et al. in rats, observed an increase in bodyweight gain during exposure to 12 h shifts twice a week (Tsai et al., 2005). This increase was only observed during the first 2 months of exposure, during the third month and a subsequent 10 day recovery period bodyweight gain was unaltered. Interestingly, in this model food intake was increased and locomotor activity was reduced which could both be linked to the observed increase in bodyweight gain. However, in contrast to the bodyweight gain, these changes were present at all time-points of the experiment.

A study by Gale et al. did not observe effects on bodyweight in rats exposed to a 6 h shift every 3 days for 10 weeks. Similar to continuous light exposure, effects of this light shift model on glucose metabolism were only observed in diabetes-prone HIP rats but not in wild type rats (increased glucose levels and decreased glucose- and arginine- stimulated insulin secretion) (Gale et al., 2011). The model used in a third study, by Bartol-Munier et al., was exposure to 10 h shifts twice a week and restriction of food to the dark phase for 5 months. In this study no effects on bodyweight were observed whether animals were on normal chow or on a high-fat diet, but changes in glucose metabolism (lower insulin levels) were present in animals fed normal chow and exposed to the shifts (Bartol-Munier et al., 2006). In the most recent study, mice were exposed to a 12 h shift once a week for 12 weeks on a normal chow diet and an additional 10 weeks on a high-fat and high-sugar diet to investigate effects on the gut microbiome. In this study a small but significant increase in bodyweight was observed in the shifted mice on a normal chow diet. When the diet was changed to a high-fat and high-sugar diet no additional effects by lighting schedule on bodyweight were observed (Voigt et al., 2014).

#### Summary models changes in light/dark schedules

Models using changes in light/dark schedules affect bodyweight in 56% (5 out of 9) of the studies, with studies using mice finding effects more often (3 out of 4) compared to studies using rats (2 out of 5). The total amount of food intake is affected in half of the studies (50%; 3 out of 6). Glucose metabolism is affected in 83% of studies (5 out of 6) with almost an equal number of studies showing an effect when using mouse or rat. These results suggest that changes in the light/dark cycle affect some of the metabolic parameters (bodyweight and glucose metabolism). For other parameters the number of studies is very low making interpretations difficult. For a complete overview of all parameters see Table 5.

## Discussion

With this review we aim to provide an overview of the available animal studies investigating the relationship between shiftwork and metabolic risk factors. Shiftwork in humans consists of a multi-aspects exposure (Figure 1). We focused on the four most relevant manipulations that are being used to mimic human shiftwork conditions in animals: altered timing of food intake, altered timing and/or duration of activity, altered timing and/or duration of sleep, and irregular lighting conditions. The overview provided in this review shows that these types of models are very useful in modeling one aspect of shiftwork and investigating the role of these separate aspects. However, the interaction between the different aspects of shiftwork is an important component of shiftwork as well, which would be beneficial to model in animals. Unfortunately, the heterogeneity of shiftwork in humans as an exposure (i.e., number of subsequent shifts, duration of recovery periods, direction of rotation, etc.) and the variability in behavioral coping responses to shiftwork amongst human individuals (for instance in sleeping and eating strategies) makes modeling shiftwork in animals a very challenging exercise. To develop an animal model that incorporates the interaction between multiple shiftwork aspects requires complete knowledge of human shiftwork behavior (i.e., light exposure, sleep behavior and dietary habits). Although in recent years first attempts have been made to achieve the latter, a complete knowledge has not been reached yet. Clearly, an animal model incorporating multiple shiftwork aspects would have advantages. Firstly, whereas human studies require over 20 years to observe long term health effects of shiftwork, such as development of metabolic disease or cancer, in an animal experiment “long-term” health effects can be studied much faster (~1 year). Hence, animal studies could accelerate the unraveling of the underlying mechanisms explaining the relationship between shiftwork and health. Secondly, animal models provide opportunities to study parameters and processes that would be impossible or extremely invasive to study in humans. In this discussion we summarize the main findings of this review, differences between models and species and touch upon possible underlying mechanisms.

### Main findings of this review

#### All categories of models

While selecting articles for this review, we came across a very diverse collection of food, activity, sleep and light manipulations which were all rather different from each other. Although we grouped the studies into four main categories, nearly none of the experimental setups was copied by another research group or was it used in a different species or strain. Furthermore, metabolic parameters were not equally frequent or extensively measured in the different models, which is another factor making it difficult to compare results between models. Five of our selected metabolic parameters (bodyweight, total food intake, total activity, glucose metabolism and lipid metabolism) were described in all four categories, but only three of them were described in both mouse and rat studies in each category. First, we will summarize the main results of all studies, followed by a discussion of the main differences between categories of models and species.

Table 6 represents the percentage of studies reporting effects of the manipulation for the listed parameters in exposed groups compared to the control group. Numerous different parameters were described in the included studies but we concentrated on a few that were measured in most studies. Bodyweight was described in most studies and 62% (26/42) of the studies reported an effect provoked by the manipulated shiftwork aspect. Total food intake and total activity levels were less often affected, in 39% (14/36) and 39% (9/23) of the studies respectively. Other metabolic parameters including energy expenditure [80% (5/6)] glucose metabolism [83% (25/30)], lipid metabolism [69% (11/16)] and adiposity [80% (8/10)] were affected frequently by shiftwork. In most studies, circadian parameters were included as well and often showed alterations (mainly in rhythm, including changes in amplitude and phase). For example, the circadian rhythm of corticosterone was altered in 54% (7/13) of the studies.

**Table 6:** **A summary of 7 most frequently measured parameters in the 5 categories of shiftwork models (food, activity, sleep, L/D shifts and LL/LDim)**.

**Model type**	**Food**	**Activity**	**Sleep**	**L/D shift**	**LL/Dim**	**All**		
	**Rat + Mouse**	**Rat + Mouse**	**Rat + Mouse**	**Rat + Mouse**	**Rat + Mouse**	**Rat**	**Mouse**	**Total**
Bodyweight	7/11 (63, 6%)	5/6 (83, 3%)	3/5 (60%)	5/9 (55, 5%)	6/11 (54, 5%)	**12/22 (54, 5%)**	**14/20 (70%)**	**26/42 (61, 9%)**
Total food intake	5/11 (45, 4%)	2/6 (33, 3%)	2/4 (50%)	3/6 (50%)	2/9 (22, 2%)	**6/16 (37, 5%)**	**8/20 (40%)**	**14/36 (38, 9%)**
Total Activity	3/7 (42, 9%)	4/6 (66, 7%)	0/3 (0%)	2/2 (100%)	0/5 (0%)	**7/11 (63, 6%)**	**2/12 (16, 7%)**	**9/23 (39, 1%)**
Total EE	3/3 (100%)		0/1 (0%)		2/2 (100%)	**1/2 (50%)**	**4/4 (100%)**	**5/6 (80%)**
Adiposity	5/7 (71, 4%)	2/2 (100%)			1/1 (100%)	**4/5 (80%)**	**4/5 (80%)**	**8/10 (80%)**
Glucose metabolism	6/9 (66, 7%)	3/3 (100%)	5/5 (100%)	5/6 (83,3%)	6/7 (85, 7%)	**13/16 (81, 2%)**	**12/14 (85, 7%)**	**25/30 (83, 3%)**
Lipid metabolism	5/7 (71, 4%)	2/2 (100%)	2/2 (100%)	1/3 (33, 3%)	1/2 (50%)	**5/7 (71, 4%)**	**6/9 (66, 7%)**	**11/16 (68, 8%)**

*The most right columns represent the results of all studies together. All results are presented as the number of studies in which an effect of the manipulation was found (when compared to the control condition)/the number of studies in which the parameter was measured. Between brackets the number of studies showing an effect is depicted as a percentage. EE, energy expenditure; L/D, light/dark; LL, continuous light; LDim, dimlight at night*.

In summary, effects on metabolism are observed in a substantial number of studies, however, results are not completely consistent. Moreover, changes in metabolism did not always translate in changes in bodyweight (gain) or adiposity. Indeed, we have to take into account that there might be a publication bias as perhaps mainly parameters that were affected are described and therefore the actual percentages of studies finding an effect might be lower.

#### Are there differences between categories?

Bodyweight is one of the parameters that was measured in all categories of models and was affected in 64% (7/11) of the food studies, in 83% (5/6) of the activity studies, in 60% (3/5) of the sleep studies, in 56% (5/9) L/D shift-studies and in 55% (6/11) of the continuous light studies. Total food intake showed to be affected in about 45% of the food studies (5/11), in 50% of sleep studies (2/4) and L/D-shift studies (3/6), whereas only 33% (2/6) of the activity-studies and 22% (2/9) of the LL-studies demonstrated an effect. Factors involved in glucose and lipid metabolism were affected in all categories of models, although light models showed low percentages for lipid metabolism (33% (1/3) in L/D shift studies; 50% (1/2) in continuous light studies). The single other parameter which was measured in all five models was total activity levels and this was affected in 43% of food studies (3/7), 67% of activity studies (4/6), 0% of sleep studies (0/3), 100% of L/D studies (2/2) and 0% of LL studies (0/5). These results show that large differences exist between the effects of different categories, however, caution is required when interpreting these results since often only a limited number of studies was available. Another important limitation to draw firm conclusions is the low number of reproducible results for many parameters. On the other hand, remarkable to notice is the 100% score for nearly each parameter measured in the studies that manipulated activity. One possible reason for this might be that 5 out of 8 of these studies came from the same research group and thereby the experimental setup was exactly the same each time, i.e., these authors produced very reproducible results. In conclusion, it is most likely that the variability between the studies (species, type of manipulation, duration of exposure etc.) is important for whether a parameter is affected by the manipulation. This is another representation of the heterogeneity of shiftwork and increases the complexity to model shiftwork. When comparing parameters and categories of models for which multiple studies are available differences are not large. As a consequence, a category with the largest metabolic consequences cannot be appointed. However, when considering human relevance of the models, the use of models using constant light and alterations in period length is least informative.

#### Are there differences between rat and mouse studies?

In the articles included for this review we observed that rats and mice are used interchangeably for shiftwork models. Interestingly, however, thus far the observed effects are not identical between species even when exactly the same procedure is carried out (Arble et al., 2009; Reznick et al., 2013). In general, in most categories either mouse (e.g., 0 out of 9 studies in activity-models) or rat studies (e.g., only 3 out of 13 studies in food-models) were underrepresented, thereby making it difficult to compare between the species. When focusing on parameters reported in at least 8 experiments in both species, neglecting the exact model category, total activity [63% (7/11)], and lipid metabolism [71% (5/7)] were more often affected in rat than in mice studies [17% (2/12), 67% (6/9) respectively]. On the other hand, effects were more often observed in mice for bodyweight [70% (14/20)], total food intake [40% (8/20)], glucose metabolism [86% (12/14)] and total energy expenditure [100% (4/4)] than in rats [55% (12/22), 38% (6/16), 81% (13/16), and 50% (1/2) respectively], however, these differences are relatively small. The only parameter showing similar percentages in both species, is adiposity with 80% (4/5) of studies showing an effect of the condition. Generally, choosing a certain type of rodent for an experiment is based on the genetic background of an animal, the similarities between the human situation/disease and the features the animal model displays, the surgical techniques that need to be carried out, the type of behavioral tests that have to be performed or other specific reasons. To this point, shiftwork models have been performed with both species and it is important to keep in mind that when creating a shiftwork model, behavioral conditions are manipulated. Often we tend to think that behavioral manipulations have similar effects in different species, but we should be aware that mice and rats may respond very differently. Causality of the dissimilar effects between mouse and rat studies is as yet unknown. Hypothetically, the difference in body size and associated metabolic rate could play a role in these differences, but this remains to be investigated.

In our opinion, an important, but lacking, model is exposure of a diurnal species to shiftwork conditions. Day-active animals are considered more similar to human when it comes to circadian research and therefore in principle would be a better model to study the metabolic consequences of shiftwork. Moreover, if similar effects on metabolism are found between nocturnal and diurnal species, this would support the translatability of animal models for human shiftwork simulations.

#### Possible mechanism of health consequences of shiftwork

Mimicking human shiftwork conditions in an animal model ultimately aims to study and understand the underlying mechanism of shiftwork leading to health problems. The predominant current theory stresses the process of desynchronization. In general, it is thought that desynchronization leads to a suboptimal functioning of many bodily processes. Observed effects range from shifts in gene expression and altered hormone secretion (i.e., leptin, insulin, melatonin and corticosterone) to modified behavioral output (i.e., food intake rhythm, activity levels and rhythm) and changes in whole body physiology (i.e., bodyweight, food intake, RER, energy expenditure, glucose and lipid metabolism). Metabolic processes within and between important metabolic tissues such as liver and muscle should cooperate in a proper timely manner to control optimally, for instance, glucose and lipid metabolism. If not, this may lead to metabolic problems.

In principal, shiftwork can cause desynchronization at different levels, which in general all result from desynchronization between the environment and the (complete circadian system within an) organism. Within the organism we distinguish 4 separate levels. The first level (1) concerns the desynchronization between the central clock and the peripheral clocks. It is well known that light is the most important Zeitgeber for the SCN, while food and activity are such for the peripheral clocks. During shiftwork these two Zeitgebers present conflicting information resulting in an opposite phase for the central and peripheral oscillators. Question is if and how these disturbances affect downstream processes.

Besides this possible top-down desynchronization between central and peripheral clocks, desynchronization may also occur between anatomically separated organs, the second level (2). Shifting the timing of food intake has been shown to differentially affect liver and muscle clocks (Bray et al., 2013; Reznick et al., 2013). Desynchronization between peripheral clocks supposedly originates from tissue-specific sensitivity to entrainment signals such as activity, energy levels (e.g., periods of fasting/feeding), responses to hormone secretion, input from autonomic nervous system etc. An additional type of desynchronization at level 2 occurs between anatomical parts of the SCN. Clock gene expression and electrical activity resynchronize differently between the dorsal and ventral part of the SCN after 6 h phase shifting (Nagano et al., 2003; Albus et al., 2005). Hypothetically, temporal desynchronization and thereby suboptimal functioning of (parts of) the SCN may lead to a malfunctioning of SCN-mediated downstream mechanisms.

The third level (3) encompasses desynchronization between the molecular clock mechanism and the clock-induced genes. Many genes involved in metabolism display a circadian rhythm in their expression. Several studies have described that a manipulation of SCN output signals, by for instance adrenalectomy or denervation of autonomic inputs, induces a loss of rhythmicity in the expression of clock-induced genes in white adipose tissue, liver and bone, while clock genes remain rhythmic (Cailotto et al., 2005, 2008; Oishi et al., 2005; Fujihara et al., 2014; Su et al., 2014). This suggests that although the molecular clock machinery is still intact, the rhythmic expression of clock-induced genes is disturbed. It is likely that this level of desynchronization indeed also takes place during shiftwork and a first suggestion was made by Salgado-Delgado et al. (2013). They showed that in their forced-activity shiftwork model the effect on the rhythmicity of metabolic genes (NAD+, Nampt, Pparα, Pparγ and Pgc1α) did not resemble the effects on clock genes rhythmicity.

The fourth level (4) of desynchronization concerns desynchronization within the molecular clock itself, i.e., different parts of the molecular clockwork are affected to a different degree within one tissue. Studies in which animals are exposed to phase shifts of the light dark cycle to induce experimental jet lag, a proper method to induce temporal circadian desynchrony, report dissimilar resynchronization speeds of different parts of the molecular clock. For instance, expression of the clock gene Cry1 appeared to resynchronize slower than mPer expression in the SCN after a 6 h phase advance (Reddy et al., 2002). Although this level of desynchronization has not yet been shown in studies using a shiftwork model, the aforementioned jetlag studies resemble studies in category 4 (i.e., shifts in timing of light exposure). Despite the body's ability to adapt to challenging conditions, this obviously becomes metabolically problematic if this occurs every few days or weeks as is the case in most working schedules of employees who are shiftworkers.

However, up to now desynchronization is mostly studied at the level of communication between central and peripheral clocks (level 1). The other three levels of desynchronization were not or only marginally studied in the aforementioned models but potentially may contribute significantly to the causal link between circadian desynchronization and negative health outcomes. Therefore, we encourage future studies to also focus on possible desynchronization at levels 2, 3, and 4.

#### Interaction of shiftwork aspects

Most studies discussed in this review used either one of four manipulations (food, activity, sleep, and light) as a model for shiftwork. Tackling shiftwork conditions by manipulating one aspect is a good approach when studying the effects of that particular aspect of shiftwork. This gives insight in how food, activity, sleep and light manipulations contribute to the associated negative health effects. However, the mentioned aspects of shiftwork are strongly intertwined and cannot easily be separated. For example, forcing an animal to consume its daily food at an unusual time inevitably also disturbs its activity and sleep pattern, which in itself also affects metabolism. Effects found of a manipulation are rapidly assigned to the main manipulation but it is often not very well considered whether and if so, how the main manipulation affects other aspects and its consequences. For instance, sleep behavior is hardly ever monitored by EEG recordings or high resolution actimetry, thus information about sleep duration and sleep quality is usually missing. In addition, in order to translate results obtained in animal studies properly to humans, also more knowledge regarding these parameters in human shiftwork is required. Thus, we propose more elaborate measurements on the main aspects of shiftwork (Figure 1) in animal as well as in human studies.

## Conclusion

This review provides an overview of animal models for shiftwork to investigate metabolic health effects. This overview indicates the large variety present in models used as well as a substantial amount of indecisive results. Ideally we would have concluded this review with suggestions for a more standardized model including a number of factors to manipulate and different possible outcome measures. Standardization would reduce the heterogeneity between studies for both methods and outcome parameters. Unfortunately, at this point our mechanistic knowledge on the effects of shiftwork is not sufficient yet to draw firm conclusions and thereby put a certain model forward or eliminate others. Furthermore, human shiftwork conditions are highly variable and not outlined well enough to propose an ideal animal model. For now, we plead for more awareness of the interactions between the aspects of shiftwork which are intentionally and unintentionally manipulated. Shiftwork and the type of manipulations used in animal models are multi-aspects exposures (Figure 1). Therefore, it is important to measure additional parameters apart from the ones directly related to the manipulation. For example, measuring sleep behavior when using a model with light shifts. Other examples are circadian parameters, such as gene expression in several organs, hormones, activity, body temperature, sleep behavior and metabolic parameters. More insights into these parameters will be beneficial for comparing different outcomes when different types of manipulations are used.

Where possible these parameters should be measured in human shiftwork studies as well to allow for more insight into translatability of findings. Furthermore, experiments ideally should cover both short- and long-term effects, ranging from days to years, to study details of underlying mechanisms in the development of the unfavorable health outcomes caused by shiftwork. The perfect model is as yet non-existent but ideally combines several aspects of shiftwork to mimic the human situation best (e.g., when manipulating activity and light, changes in food intake will follow and this should be monitored).

Only by properly studying the effects of shiftwork conditions solely and combined, this research eventually will help the general community to learn how to deal with shiftwork conditions best, prevent shiftworkers from becoming disturbed and possibly prevent and treat negative health outcomes.

### Conflict of interest statement

The authors declare that the research was conducted in the absence of any commercial or financial relationships that could be construed as a potential conflict of interest.
