# Supplementary material for: Rodent models to study the metabolic effects of shiftwork in humans
Source: Front Pharmacol. 2015 Mar 24;6:50. doi: 10.3389/fphar.2015.00050 (PMC4371697; doi:10.3389/fphar.2015.00050)
Supplement: Supplementary file 1 [file DataSheet1.DOCX]

***Supplementary Material***

**Rodent models to study the metabolic effects of shiftwork in humans**

**Anne-Loes Opperhuizen_1_^1#^, Linda W. M. van Kerkhof_1_^2#^, Karin I. Proper_2_^2^, Wendy Rodenburg_3_^2^, Andries Kalsbeek_4_^1,3*^**

^1^ Netherlands Institute for Neuroscience, Hypothalamic Integration Mechanisms, Amsterdam, the Netherlands
^2^ National Institute for Public Health and the Environment, Bilthoven, The Netherlands
^3^ Academic Medical Center (AMC), Department of Endocrinology and Metabolism, University of Amsterdam, the Netherlands

^#^ these authors contributed equally to this review

*** Correspondence:** Prof. Dr. Andries Kalsbeek, Academic Medical Center Amsterdam, Department Endocrinology and Metabolism, University of Amsterdam, Meibergdreef 9, 1105 AZ Amsterdam, The Netherlands
[a.kalsbeek@amc.uva.nl](mailto:a.kalsbeek@amc.uva.nl)

**1. Supplementary Data**

**Search Strategy**

Database: MEDLINE 1950 to present, MEDLINE In-Process & Other Non-Indexed Citations

Search Strategy: 31-07-2013

--------------------------------------------------------------------------------

1 (shift work* or shiftwork* or night work* or nightwork* or night shift* or nightshift* or rotating shift* or early shift* or late shift* or jet lag or jetlag or working rhythm* or "irregular working hours" or "atypical work hours " or (light-phase and restricted) or ("phase restricted" and light) or time restricted or "constant light" or "continuous light" or "light at night" or "light-dark cycle").ti. (2847)

2 (working conditions or work shift*).ti. and (shift work* or shiftwork* or night work* or nightwork* or night shift* or nightshift* or rotating shift* or early shift* or late shift* or jet lag or jetlag or working rhythm* or "24/7" or "irregular working hours" or " atypical work hours").tw. (65)

3 (biological clock*OR body clock* or "internal clock system" or chronobiolog* or circadian clock* or (circadian adj5 rhythm*) or (circadian adj5 cycle*) or circadian control or circadian biology or circadian disruption or chronodisruption or circadian desynchron* or circadion dysfunct* or (circadian adj5 metabol*) or circadian metabolome or circadian timing or (circadian adj5 pattern*) or abnormal circadian or circadian adjustment or clock genes or clock-controlled genes or circadian gene clock*).ti. (11932)

4 1 or 2 or 3 (14583)

5 (weight or body weight or weight change or weight increase or weight gain or gain weight or weight regulation or overweight or body mass index or body mass control or metabolic syndrome or obesity or adiposity or metabolic processes or metabolic pathways or metabolic rhythm*OR metabolic disease*OR metabolic disturbance or metabolic disruption or metabolic response* or metabolic desynchrony or metabolic dissynchrony or metabolic dysfunction* or metabolic derangement* or metabolic health or metabolic function* or metabolic consequences or metabolic assessment or metabolic genes or metabolome).ti. (153243)

6 (metabolism or digestion or glucose or glucose intolerenace or glucose tolerance or lipid metabolism or energy metabolism or energy balance or feeding metabolism or nutritional processes or insulin or insulin sensitivity or insulin resistance or insulin tolerance or insulin secretion or beta cell function or blood sugar or serum cholesterol or cardiometabolic or blood pressure or arterial pressure or heart rate or blood lipids or hypertension or cholesterol or leptin or ghrelin or orexin).ti. (594162)

7 *body weight/ or *weight gain/ or *weight loss/ or *overweight/ or *body mass index/ or *metabolism/ or *energy metabolism/ or *metabolic syndrome X/ or *metabolic diseases/ or *obesity/ or *adiposity/ or *glucose/ or *blood glucose/ or *glucose metabolism disorders/ or *glucose tolerance test/ or *hyperglycemia/ or *hypoglycemia/ or *insulin/ or *insulin resistance/ or *hyperlipidemias/ or *dyslipidemias/ or *blood pressure/ or *hypertension/ or *nutritional physiological phenomena/ or *intra-abdominal fat/ or exp *cholesterol/ or *leptin/ or *ghrelin/ (633472)

8 (physiolog* or health or mortality).ti. (561527)

9 (weight or body weight or weight change or weight increase or weight gain or gain weight or weight regulation or overweight or body mass index or body mass control or metabolic syndrome or obesity or adiposity or metabolic processes or metabolic pathways or metabolic rhythm*OR metabolic disease*OR metabolic disturbance or metabolic disruption or metabolic response* or metabolic desynchrony or metabolic dissynchrony or metabolic dysfunction* or metabolic derangement* or metabolic health or metabolic function* or metabolic consequences or metabolic assessment or metabolic genes or metabolome).tw. (775903)

10 (metabolism or digestion or glucose or glucose intolerenace or glucose tolerance or lipid metabolism or energy metabolism or energy balance or feeding metabolism or nutritional processes or insulin or insulin sensitivity or insulin resistance or insulin tolerance or insulin secretion or beta cell function or blood sugar or serum cholesterol or cardiometabolic or blood pressure or arterial pressure or heart rate or blood lipids or hypertension or cholesterol or leptin or ghrelin or orexin).tw. (1508391)

11 8 and (9 or 10) (47347)

12 4 and (5 or 6 or 7 or 11) (1518)

13 (cardiovascular or cardiometabolic or cvd or diabetes or diabetic or chronic disease*).ti. (312355)

14 exp *cardiovascular diseases/ or *cardiovascular physiological processes/ or *cardiovascular physiological phenomena/ or *cardiovascular system/ or exp *diabetes mellitus/ or *diabetes mellitus, type 2/ or *chronic disease/ (1773251)

15 4 and (13 or 14) (967)

16 12 or 15 (2005)

17 16 and (english or dutch or german).lg. (1690)

18 limit 17 to yr=1993-2013 (1231)

19 remove duplicates from 18 (1111)

20 ((blood pressure or hypertens*) and (treatment or therap* or administration)).ti. (25611)

21 ("in vitro" or "in vivo" or pacemaker* or drosophila or circadian blood pressure).ti. (403442)

22 (circadian and (myocardial or infarction)).ti. (214)

23 19 not (20 or 21 or 22) (984)

***************************

Legend OvidSP search commands:

tw search in title or abstract

ti search title only

/ search as keyword (MeSH)

*keyword/ search as major keyword (major MeSH)

hw search as word in keyword field

/ep field keyword, in this case epidemiology

exp searched as keyword including related keywords

fs searched in keyword field as subheading of keywords

? one or no sign

adj3 max. 2 words between search terms

lg language

pt publication type

rn searched in CAS-nr field

* truncation sign

4 databases with 87439777 documents selected

ME90 MEDLINE NLM (for excluding doubles)

EM90 EMBASE 2013 Elsevier B.V.

BA70 BIOSIS Previews Thomson Reuters

IS74 SciSearch Thomson Reuters

Search Strategy: Date: 07-08-2013

NO HITS SEARCH EXPRESSION

-- ----- ---------- --------------------------------------------------------

C= 1 87439777 ME90; EM90; BA70; IS74

S= 2 7496 FT=(SHIFT WORK*;SHIFTWORK*;NIGHT WORK*;NIGHTWORK*;NIGHT

SHIFT*;NIGHTSHIFT*;ROTATING SHIFT*;EARLY SHIFT*;LATE

SHIFT*;JET LAG;JETLAG;WORKING RHYTHM*;IRREGULAR WORKING

HOURS;ATYPICAL WORK HOURS;TIME RESTRICTED)/TI

3 2444 FT=(CONSTANT LIGHT;CONTINEOUS LIGHT;LIGHT AT

NIGHT;LIGHT-DARK CYCLE)/TI OR (FT=(LIGHT-PHASE)/TI AND

FT=(RESTRICTED)/TI) OR (FT=(PHASE RESTRICTED) AND

FT=(LIGHT)/TI)

4 183 FT=(WORKING CONDITIONS;WORK SHIFT*)/TI AND FT=(SHIFT

WORK*;SHIFTWORK*;NIGHT WORK*;NIGHTWORK*;NIGHT

SHIFT*;NIGHTSHIFT*;ROTATING SHIFT*;EARLY SHIFT*;LATE

SHIFT*;JET LAG;JETLAG;WORKING RHYTHM*;IRREGULAR WORKING

HOURS;ATYPICAL WORK HOURS)/(TI;AB;UT)

5 43154 FT=(BIOLOGICAL CLOCK*;BODY CLOCK*;"INTERNAL CLOCK

SYSTEM";CHRONOBIOLOG*;CIRCADIAN CLOCK*;CIRCADIAN # # # #

# RHYTHM*;CIRCADIAN # # # # # CYCLE*;CIRCADIAN

CONTROL;CIRCADIAN BIOLOGY;CIRCADIAN

DISRUPTION;CHRONODISRUPTION;CIRCADIAN DESYNCHRON*)/TI

6 5024 FT=(CIRCADION DYSFUNCT*;CIRCADIAN # # # # #

METABOL*;CIRCADIAN METABOLOME;CIRCADIAN TIMING;CIRCADIAN

# # # # # PATTERN*;ABNORMAL CIRCADIAN;CIRCADIAN

ADJUSTMENT;CLOCK GENES;CLOCK-CONTROLLED GENES;CIRCADIAN

GENE CLOCK*)/TI

7 56074 2 OR 3 OR 4 OR 5 OR 6

8 578049 FT=(WEIGHT;BODY WEIGHT;WEIGHT CHANGE;WEIGHT

INCREASE;WEIGHT GAIN;GAIN WEIGHT;WEIGHT

REGULATION;OVERWEIGHT;BODY MASS INDEX;BODY MASS

CONTROL;METABOLIC SYNDROME;OBESITY;ADIPOSITY;METABOLIC

PROCESSES;METABOLIC PATHWAYS;METABOLIC RHYTHM*)/TI

9 17669 FT=(METABOLIC DISEASE*;METABOLIC DISTURBANCE;METABOLIC

DISRUPTION;METABOLIC RESPONSE*;METABOLIC

DESYNCHRONY;METABOLIC DYSSYNCHRONY;METABOLIC

DYSFUNCTION*;METABOLIC DERANGEMENT*;METABOLIC

HEALTH;METABOLIC FUNCTION*)/TI

10 839760 FT=(METABOLIC CONSEQUENCES;METABOLIC

ASSESSMENT;METABOLIC

GENES;METABOLOME;METABOLISM;DIGESTION;GLUCOSE;GLUCOSE

INTOLERANCE;GLUCOSE TOLERANCE;LIPID METABOLISM;ENERGY

METABOLISM;ENERGY BALANCE;FEEDING METABOLISM;NUTRITIONAL

PROCESSES)/TI

11 470374 FT=(INSULIN;INSULIN SENSITIVITY;INSULIN

RESISTANCE;INSULIN TOLERANCE;INSULIN SECRETION;BETA CELL

FUNCTION;BLOOD SUGAR;SERUM CHOLESTEROL)/TI

12 815373 FT=(CARDIOMETABOLIC;BLOOD PRESSURE;ARTERIAL

PRESSURE;HEART RATE;BLOOD

LIPIDS;HYPERTENSION;CHOLESTEROL;LEPTIN;GHRELIN;OREXIN)/T

I

13 252048 CT=(BODY WEIGHT;WEIGHT GAIN;BODY WEIGHT GAIN;WEIGHT

LOSS;WEIGHT REDUCTION;OVERWEIGHT;BODY MASS

INDEX;METABOLISM;ENERGY METABOLISM;METABOLIC SYNDROME

X;METABOLIC DISEASES;METABOLIC BALANCE;METABOLIC

DISORDER;METABOLIC REGULATION)/W=1

14 286924 CT=(OBESITY;ADIPOSITY;GLUCOSE;BLOOD GLUCOSE;GLUCOSE

BLOOD LEVEL;GLUCOSE METABOLISM DISORDERS;GLUCOSE

TOLERANCE TEST;GLUCOSE TOLERANCE;GLUCOSE

METABOLISM;HYPERLIPIDEMIA;DYSLIPIDEMIA;INSULIN

METABOLISM;ABDOMINAL FAT)/W=1

15 510593 CT=(HYPERGLYCEMIA;HYPOGLYCEMIA;INSULIN;INSULIN

RESISTANCE;HYPERLIPIDEMIAS;DYSLIPIDEMIAS;BLOOD

PRESSURE;HYPERTENSION;NUTRITIONAL PHYSIOLOGICAL

PHENOMENA;INTRA-ABDOMINAL FAT;LEPTIN;GHRELIN)/W=1 OR CT

D CHOLESTEROL/W=1

16 1468120 FT=(PHYSIOLOG*;HEALTH;MORTALITY)/TI

17 3019707 FT=(WEIGHT;BODY WEIGHT;WEIGHT CHANGE;WEIGHT

INCREASE;WEIGHT GAIN;GAIN WEIGHT;WEIGHT

REGULATION;OVERWEIGHT;BODY MASS INDEX;BODY MASS

CONTROL;METABOLIC SYNDROME;OBESITY;ADIPOSITY;METABOLIC

PROCESSES;METABOLIC PATHWAYS)/(TI;AB;UT)

18 105828 FT=(METABOLIC DISEASE*;METABOLIC DISTURBANCE;METABOLIC

DISRUPTION;METABOLIC RESPONSE*;METABOLIC

DESYNCHRONY;METABOLIC DYSSSYNCHRONY;METABOLIC

DYSFUNCTION*;METABOLIC DERANGEMENT*;METABOLIC

HEALTH;METABOLIC FUNCTION*)/(TI;AB;UT)

19 2870436 FT=(METABOLIC RHYTHM*;METABOLIC CONSEQUENCES;METABOLIC

ASSESSMENT;METABOLIC

GENES;METABOLOME;METABOLISM;DIGESTION;GLUCOSE;GLUCOSE

INTOLERANCE;GLUCOSE TOLERANCE;LIPID METABOLISM;ENERGY

METABOLISM;ENERGY BALANCE)/(TI;AB;UT)

20 1096395 FT=(INSULIN;INSULIN SENSITIVITY;INSULIN

RESISTANCE;INSULIN TOLERANCE;INSULIN SECRETION;BETA CELL

FUNCTION;BLOOD SUGAR;SERUM CHOLESTEROL)/(TI;AB;UT)

21 2435118 FT=(FEEDING METABOLISM;NUTRITIONAL

PROCESSES;CARDIOMETABOLIC;BLOOD PRESSURE;ARTERIAL

PRESSURE;HEART RATE;BLOOD

LIPIDS;HYPERTENSION;CHOLESTEROL;LEPTIN;GHRELIN;OREXIN)/(

TI;AB;UT)

22 175859 16 AND (17 OR 18 OR 19 OR 20 OR 21)

23 5517 7 AND (8 OR 9 OR 10 OR 11 OR 12 OR 13 OR 14 OR 15 OR 22)

24 1084405 FT=(CARDIOVASCULAR;CARDIOMETABOLIC;CVD;DIABETES;DIABETIC

;CHRONIC DISEASE*)/TI

25 2652825 CT D (CARDIOVASCULAR DISEASES;CARDIOVASCULAR

DISEASE;DIABETES MELLITUS)/W=1 OR CT=(CARDIOVASCULAR

PHYSIOLOGICAL PROCESSES;CARDIOVASCULAR PHYSIOLOGICAL

PHENOMENA;CARDIOVASCULAR FUNCTION;CARDIOVASCULAR

SYSTEM)/W=1

26 152991 CT=(DIABETES MELLITUS, TYPE 2;CHRONIC DISEASE)/W=1

27 2260 7 AND (24 OR 25 OR 26)

28 6705 23 OR 27

29 6087 28 AND LA=(ENGLISH;DUTCH;GERMAN)

30 4794 29 AND PY>1992

31 69864 FT=(BLOOD PRESSURE;HYPERTENS*)/TI AND

FT=(TREATMENT;THERAP*;ADMINISTRATION)/TI

32 1396417 FT=(IN VITRO;IN VIVO;PACEMAKER*;DROSOPHILA;CIRCADIAN

BLOOD PRESSURE)/TI

33 866 FT=CIRCADIAN/TI AND FT=(MYOCARDIAL;INFARCTION)/TI

34 7479002 DT=MEETING ABSTRACT

35 3701 30 NOT (31 OR 32 OR 33 OR 34)

36 1590 check duplicates: unique in s=35

37 1024 36 AND BASE=ME90

38 566 36 NOT 37

**************************************

Legenda DIMDI search-commands:

f = find

ft = free term

ct = controlled term (=keyword)

ct d = controlled term (keyword) with all underlying keywords

ut = uncontrolled term (in keyword-field)

/W=1 = controlled term searched in the major keyword-field

* = truncationsign: stem word + all possible endings

/ti = searched in titel

/(ti;ab)= searched in titel or abstract)

# # # = neighbourhood searching (max 3 words between the 2 search terms)

dt = document type

la = language
